# Cross-Linked Gamma Polyglutamic Acid/Human Hair Keratin Electrospun Nanofibrous Scaffolds with Excellent Biocompatibility and Biodegradability

**DOI:** 10.3390/polym14245505

**Published:** 2022-12-15

**Authors:** Ming Hao, Yanbo Liu, Zhijun Chen, Xiaodong Hu, Tianyi Zhang, Xinyu Zhu, Xingyu He, Bo Yang

**Affiliations:** 1 State Key Laboratory of New Textile Materials and Advanced Processing Technologies, School of Textile Science and Engineering, Wuhan Textile University, Wuhan 430200, China; 2 State Key Laboratory of Separation Membranes and Membrane Processes, School of Textile Science and Engineering, Tiangong University, Tianjin 300387, China

**Keywords:** electrospun nanofibrous scaffolds, γ-PGA, human hair keratin, biocompatibility, biodegradability

## Abstract

Recently, human hair keratin has been widely studied and applied in clinical fields due to its good histocompatibility, biocompatibility, and biodegradability. However, the regenerated keratin from human hair cannot be electrospun alone because of its low molecular weight. Herein, gamma polyglutamic acid (γ-PGA) was first selected to fabricate smooth and uniform γ-PGA/keratin composite scaffolds with excellent biocompatibility and biodegradability by electrospinning technology and a chemical cross-linking method in this study. The effect of electrospinning parameters on the structure and morphology, the mechanism of chemical cross-linking, biocompatibility in vitro cell culture experiments, and biodegradability in phosphate-buffered saline buffer solution and trypsin solution of the γ-PGA/keratin electrospun nanofibrous scaffolds (ENS) was studied. The results show that the cross-linked γ-PGA/keratin ENSs had excellent water stability and biodegradability. The γ-PGA/keratin ENSs showed better biocompatibility in promoting cell adhesion and cell growth compared with the γ-PGA ENSs. It is expected that γ-PGA/keratin ENSs will be easily and significantly used in tissue engineering to repair or regenerate materials.

## 1. Introduction

Human hair keratin is a natural, hard, and fibrous protein with connective tissue and protective functions. It is derived from the human body itself and composed of water-insoluble polypeptide chains in parallel with the α-helix, β-pleated sheet, and random coil forms. There is no antigenicity (immune response) upon implantation owing to the excellent histocompatibility, biocompatibility, and biodegradability of purified samples. Furthermore, it also contains the cell-binding motif Leu-Asp-Val [1], which promotes the adhesion of α4β1-integrin-mediated human dermal fibroblasts (HDFs) [2] and lymphoid cells [3], resulting in less immune rejection and excellent tissue regeneration with the use of keratin scaffolds.

Electrospun nanofiber membranes (ENMs) have been widely used in filtration [4,5,6,7,8,9], sensors [10,11], battery components [12,13,14], waterproof and breathable membranes [15,16], wearable textile devices [17,18,19], and so on due to their outstanding advantages, such as a high specific surface area, high porosity, good fiber uniformity, and controllable fiber accumulation. In particular, ENMs are also widely used in tissue engineering owing to their non-porous and 3D structure, which is similar to the extracellular matrix [20,21,22,23,24,25,26]. However, the regenerated keratin from human hair cannot be electrospun alone because the molecular weight of the extracted keratin is too low. Therefore, it has to be electrospun together with synthetic polymers to improve the spinnability of the keratin solution. A keratin ENM with other polymers has good histocompatibility, biocompatibility, and biodegradability and could be used as an alternative template for tissue regeneration, wound dressings, tissue engineering scaffolds [27,28], etc.

In general, polycaprolactone (PCL), polylactic acid (PLA), and polyhydroxy butyrate (PHB) are the most popular synthetic polymers for fabricating the corresponding scaffolds. However, there remain some limitations when these polymers are used in practical work. With the disadvantage of hydrophobicity, PCL has poor wettability, low cell adhesion, and a slow degradation rate [29]. The poor mechanical properties and hydrophilicity of PLA impair cell adhesion and differentiation [30]. PHB has insufficient cell affinity, which may accompany some limitations [30]. Therefore, it is necessary to use a biocompatible polymer with good hydrophilicity to further improve the biocompatibility of keratin/polymer scaffolds. Gamma polyglutamic acid (γ-PGA) is an extracellular viscous substance produced by several bacteria belonging to the genus Bacillus and belongs to a naturally occurring polyamine acid [31]. It has a broad range of applications in biomedicine and other fields due to its excellent water solubility, biocompatibility, and biodegradability [32,33,34,35,36,37]. Since γ-PGA ENMs and keratin/polymer scaffolds have been prepared successfully, it is assumed that the creation of electrospun nanofiber scaffolds (ENSs) composed of γ-PGA and keratin will be possible and become more important in the biomedical field. To the best of our knowledge, no report has been published on such γ-PGA/keratin ENSs. In this study, γ-PGA/keratin ENSs were successfully constructed by electrospinning under optimized parameters for the first time. In order to improve the stability of ENSs in PBS, 1-(3-Dimethylaminopropyl)-3-ethyl carbodiimide hydrochloride (EDC)/*n*-hydroxy succinimide (NHS)/cystamine was used for the cross-linking treatment of ENSs. The cross-linked NHSs had good stability and a good swelling rate (greater than 320%) in PBS. In addition, benefitting from the excellent biocompatibility and biodegradability of the materials, the prepared ENSs also had good biocompatibility and controllable biodegradation. We believe that the γ-PGA/keratin NHS is a promising candidate material for tissue engineering.

## 2. Materials and Methods

### 2.1. Materials

The γ-PGA (Mw = 700 kDa) was purchased from EKEAR Bio@Tech Co., Ltd. (Shanghai, China). Trifluoroacetic acid (TFA), 1-(3-Dimethylaminopropyl)-3-ethyl carbodiimide hydrochloride (EDC), cystamine, chloroform, and methanol were purchased from Aladdin Bio-Chem Technology Co., Ltd. (Shanghai, China). The *n*-hydroxy succinimide (NHS) was purchased from Macklin Biochemical Co., Ltd. (Shanghai, China). Phosphate-buffered saline (PBS) solution was purchased from Aspen Biotechnology Co., Ltd. (Wuhan, China). Bottled trypsin was purchased from Google Biotech Co., Ltd. (Wuhan, China). The CCK-8 kit was purchased from Saiguo Biotech Co., Ltd. (Guangzhou, China). The murine fibroblasts (L929) were purchased from the Cell Bank of the Chinese Academy of Sciences (Shanghai, China). The human hair was obtained from a common barbershop for Chinese people.

### 2.2. Preparation of Regenerated Human Hair Keratin

The human hair was washed with soapy water. Then, the washed hair was degreased by immersing it in a mixed solution of chloroform and methanol (2:1, *v*/*v*) for 24 h [38]. The hair was then dried and cut into small pieces (1 cm). The defatted hair fragments (20 g) were put into 400 mL of hydrochloric acid solution (6 M) and treated at 110 °C for 9 h. After filtering and removal of the undissolved hair, the pH value of the filtrate was adjusted to neutral and insoluble substances were removed by centrifuging the filtrate for 5 min. The centrifuged solution was poured into a dialysis bag (12–14 kDa) and dialyzed against deionized water for 3 days, during which the water was replaced every 12 h. The centrifuged keratin solution was freeze-dried to obtain solid, regenerated human hair keratin.

### 2.3. Preparation of γ-PGA/Keratin ENSs

A certain mass of γ-PGA and keratin was dissolved in TFA aqueous solution and stirred sufficiently at room temperature to obtain γ-PGA and γ-PGA/keratin spinning solution. The prepared solution was injected into a syringe equipped with a 20# needle. A high-voltage power supplier was connected to the needle, and the metal roller of the receiving device was grounded. The spinning parameters were set as follows: the concentration was 24~30%, the γ-PGA/keratin ratio was 9:1~6:4, the spinning voltage was 36 kV~42 kV, and the receiving distance was 18 cm~24 cm. The optimum parameters were determined according to the spinning process and the uniformity of the ENS fibers. The prepared ENSs under optimized parameters were stored in a vacuum desiccator for subsequent analysis. In addition, the relative humidity was held below 30% during the electrospinning process due to the γ-PGA’s strong hygroscopicity. 

### 2.4. Preparation of Cross-Linked γ-PGA/Keratin ENSs

A solution of 1-(3-Dimethylaminopropyl)-3-ethyl carbodiimide hydrochloride (EDC)/*n*-hydroxy succinimide (NHS)/cystamine in methanol (*n* (EDC)/*n* (NHS) = 1:1) was prepared, wherein the concentration of EDC was 4.5 wt% and the concentration of cystamine in the solution was 2 mg/mL. Then, γ-PGA/keratin ENSs were cut into 5×5 cm^2^ specimens and immersed in the above solution for 12 h, 24 h, 36 h, 48 h, and 60 h, respectively. The remaining cross-linking agent was washed off the membranes by using methanol solution. After freeze-drying at −50 °C for 48 h, the cross-linked γ-PGA/keratin ENSs were obtained and stored in a vacuum desiccator (DZF-6020, Yuhua Instrument Co., Ltd., Gongyi, China). The hydrolysis-resistant γ-PGA ENSs were treated according to the method reported in [39].

### 2.5. Characterization

The morphology of ENSs was characterized by an SEM (JSM-IT300A, Japan Electronics Co., Ltd., Tokyo, Japan) with an acceleration voltage of 20 kV and a working distance of about 20 mm. The release paper with the ENSs was cut into small pieces of 1 cm × 1 cm and fixed on an electron microscope stage with double-sided tape. The sample was sputtered in a thin argon atmosphere using a fully automatic ion-sputtering apparatus (JEC-3000FC, Japan Electronics Co., Ltd., Tokyo, Japan) for 180 s. The fiber diameter was measured by Image ProPlus software. At least 50 nanofibers from each sample were randomly selected and analyzed, and the average diameter and standard deviation were calculated. The groups of γ-PGA/keratin membranes before and after cross-linking were analyzed by a Fourier Transform Infrared (FTIR) Spectrometer (Tensor 27, Bruker Corporation, Saarbrucken, Germany).

The weight loss ratios and swelling degrees for both cross-linked and uncross-linked ENSs were calculated after preparation for different cross-linking durations. The electrospun samples were first dried and then weighed, and the weighing results were recorded as *W*_0_. The ENSs were later immersed in PBS, which was heated at 37 °C in a water bath. The samples were taken out of the PBS in the water bath after treatment for 48 h and then placed on a glass dish with a tilt angle of 60°. The samples were weighed and recorded as *W*_1_ when no water was observed to flow out of the samples. Then, the samples were dried to a constant weight and weighed as *W*_2_. The swelling degrees (*S*) and the weight loss ratios (*W*) of the ENSs were calculated by the following formula:

(1) $$S=\frac{W_{1}-W_{0}}{\rho \times W_{0}}\times 100\%$$

(2) $$W=\frac{W_{0}-W_{2}}{W_{0}}\times 100\%$$

A TGA/DSC 1 Synchronous Thermal Analyzer (TGA/DSC 1, METTLER TOLEDO, Co., Ltd., Zurich, Switzerland) was used to test and analyze the thermal decomposition temperatures of the electrospun samples before and after cross-linking. The samples were tested in a nitrogen atmosphere at elevated temperatures with a gradient of 10 °C/min in the range from 50 °C to 900 °C. The tensile properties of the membranes at different cross-linking times were measured by a universal tensile testing machine (LTD E44.104, MTS Systems Corporation, Eden Prairie, MN, USA). Notably, wet conditions were simulated by soaking the samples in PBS for 5 min and then removing the surface moisture with filter paper. The samples were cut into 30 mm × 10 mm pieces. The stretching speed was set to 20 mm min^−1^ and the test was continued until the membrane broke apart.

The γ-PGA ENSs were cut into square specimens (20 mm × 20 mm), dried, and then weighed and recorded as *G*_0_. Membranes were separately immersed in phosphate-buffered saline and phosphate-buffered saline containing trypsin (10 mg/mL) at 37 °C for 1, 2, 3, and 4 weeks. We refreshed the solution every three days, removed the membranes from the solution for a specified period, rinsed them with distilled water, dried them, and then weighed them as *G*_1_. The morphology of the fiber membranes was observed by SEM. The weight loss rate *L* of the fiber membranes was obtained by the following equation:

(3) $$L=\frac{G_{0}-G_{1}}{G_{0}}\times 100\%$$

The fiber membranes were made into small discs of 6 mm in diameter with a puncher, and each group of 3 samples was immersed in 75% alcohol for 1 h. After being exposed to both sides of a UV lamp for 0.5 h, the cells were immersed 3 times in PBS and plated on a 96-well culture plate. A total of 100 μL of the L929 cell suspension (1 × 10^4^ cells/mL) was seeded on a 96-well culture plate, and the unreleased group was set as a control group. The 96-well culture plate inoculated with the cells was then placed in a 5%CO_2_ incubator (CCL-170B-8, Esco Micro Pte. Ltd., Singapore) and incubated at 37 °C for 1 d, 3 d, and 5 d, respectively. The culture solution was refreshed every 2 d to maintain the freshness and effects of the nutritious components.

The γ-PGA/keratin membranes and the γ-PGA membranes inoculated with cells for 3 days were immersed 3 times in PBS buffer. Subsequently, they were fixed with 4% glutaraldehyde at a temperature of 4 °C for 2 h, then washed 3 times with PBS. The gradient dehydration treatment was carried out with different concentrations of ethanol. The dehydrated fiber membranes were freeze-dried, and the growth of the cells was observed by SEM.

The ENSs were taken out at the corresponding time and immersed 3 times in PBS. A total of 100 mL of fresh complete medium was added to each well. Then, 10 mL of Cell Counting Kit-8 (CCK-8) reagent was added, and the plate was gently tapped to help the CCK-8 reagent mix. After incubation for 2 h at 37 °C in a 5% CO_2_ incubator, the absorbance at 450 nm was measured with an enzyme labeling instrument (Infinite^®^ 200 Pro, TECAN, Mannedorf, Switzerland).

The cell culture samples seeded for 1 d were washed once with 1× Assay Buffer and incubated for 15 min at 37 °C in 2 μM Calcein-AM and 4.5 μM PI in 1× Assay Buffer. Viable cells (cells with a yellow-green fluorescence) and dead cells (cells with a red fluorescence) were simultaneously detected using a 490 ± 10 nm filter under a fluorescence microscope.

### 2.6. Statistical Analysis

Results are shown as mean ± standard deviation. Statistical analysis was performed by the analysis of variance (ANOVA) method. A value of *p* < 0.05 was considered statistically significant.

## 3. Results

### 3.1. Optimization of Process Parameters of γ-PGA/Keratin Nanofibers

Electrospinning parameters have an important influence on the morphology of electrospinning nanofibers. Shige Wang et al. reported that uniform nanofibers can be prepared by dissolving γ-PGA powder in 1–10 wt% TFA aqueous solution [40]. Inspired by this, we found that the keratin and γ-PGA mixed in TFA aqueous solution could be co-electrospun successfully to generate nanofibers. Additionally, parameters such as the ratio of keratin to γ-PGA, the solution concentration, the voltage applied, and the receiving distance were systematically studied to obtain nanofibers with a uniform diameter distribution.

**Figure 1:**
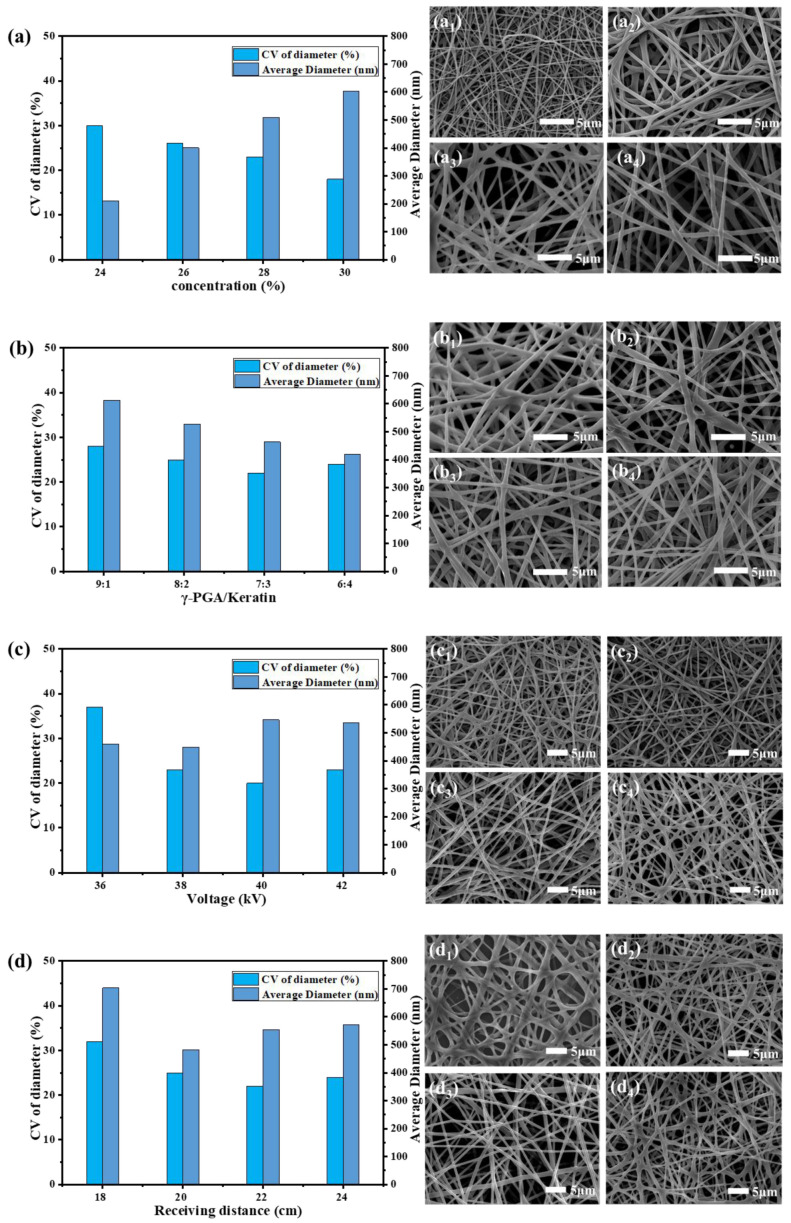
Average diameter, CV value of the diameter, and SEM images of γ-PGA/keratin ENSs under different spinning parameters. (**a**) Concentrations (**a_1_**~**a_4_** represent SEM images of ENSs at a spinning solution concentration of 24%, 26%, 28%, and 30%, respectively). (**b**) γ-PGA/keratin ratios (**b_1_**~**b_4_** represent SEM images of ENSs at a γ-PGA/keratin ratio of 9:1, 8:2, 7:3, and 6:4, respectively). (**c**) Voltages (**c_1_**~**c_4_** represent SEM images of ENSs at a voltage of 36 kV, 38 kV, 40 kV, and 42 kV, respectively). (**d**) Receiving distance (**d_1_**~**d_4_** represent SEM images of ENSs at a receiving distance of 18 cm, 20 cm, 22 cm, and 24 cm, respectively).

In our research, we found that when the concentration of the γ-PGA/keratin mixture solution was less than 23 wt%, the surface tension of the solution was too low to maintain a stable “Taylor cone” at the tip of the syringe during the spinning process. The “Taylor cone” was stably present while the concentration of the spinning solution was 24 wt% or more. As can be seen from Figure 1a, as the solution concentration increased from 24 wt% to 30 wt%, the average diameter of the obtained fiber increased from 211 nm to 604 nm and the fiber became more uniform. However, the needle was easily blocked during the spinning process when the solution concentration was 30 wt% or higher, which affected the yield.

It can be seen from Figure 1b that, as the keratin content increased, the average diameter of the ENS gradually became smaller because the molecular weight of the keratin was much lower than the molecular weight of the γ-PGA. The content of γ-PGA and the viscosity of the solution decreased as the keratin content increased in the mixed solution, which resulted in a decrease in the average diameter of the fibers. 

Figure 1c shows that the nanofibers can be obtained at voltages of 36 kV to 42 kV. As can be seen in Figure 1d, the solvent had not yet fully evaporated when the receiving distance was 18 cm, resulting in an increase in the average diameter of the fibers. As the receiving distance increased, the average diameter of the fibers became larger because the electric field’s strength decreased as the receiving distance was further increased. The tensile action of the jet in the electric field was weakened.

In summary, the optimal operating parameters for preparing smooth and uniform γ-PGA/keratin nanofibers were as follows: a polymer concentration of 28 wt%, a γ-PGA/keratin ratio of 7:3, an applied voltage of 40 kV, a collection distance of 22 cm, and an environmental humidity of less than 30%. Smooth and uniform γ-PGA/keratin nanofibers with a diameter of 554 ± 73 nm were successfully prepared under these conditions.

### 3.2. Principle of Cross-Linking

**Figure 2:**
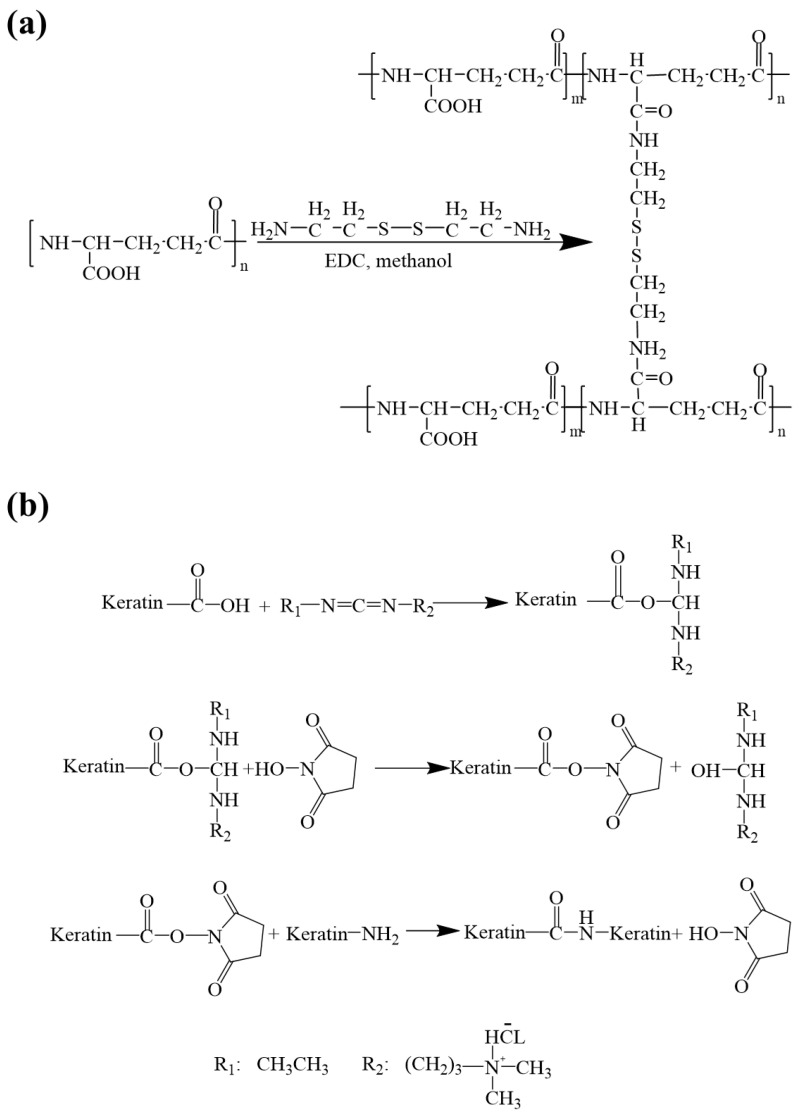
Schematic of the cross-linking reaction: (**a**) cross-linking between γ-PGA and cystamine molecules; (**b**) cross-linking between the keratin and the γ-PGA.

In order to obtain water-stable membranes, the membranes were cross-linked by using EDC/NHS/cysteamine to form amide bonds between the amino group and the carboxyl group. The principle of the fiber membrane cross-linking treatment is illustrated in Figure 2.

The scaffold is composed of two materials, namely γ-PGA and keratin. As γ-PGA contains a large number of hydrophilic groups, it is easily soluble in water. The function of EDC is to activate the carboxyl group in the polyglutamic acid so that the amino group in the cysteamine will react with the hydrophilic group in the polyglutamic acid to form an amide bond [39,40]. The specific reaction is shown in Figure 2a.

The cross-linking principle of keratin hydrolysis resistance is shown in Figure 2b. As a commonly used cross-linking agent for biomaterials, EDC only helps the amino and carboxyl groups of protein molecules dehydrate and condense to form amide bonds during the reaction.

The EDC is first conjugated with the carboxyl group to form a urea derivative, which will form an amide bond with the -NH_2_ group and can be cleaned off. NHS enhances the stability of carbon diamide cross-linked products by forming more stable esters. In the cross-linking process, EDC and NHS are not part of the actual cross-linking, so this cross-linking treatment will not make the raw materials very cytotoxic [41]. The mechanism of cross-linking is that the amino and carboxyl groups of keratins dehydrate and condense to form amide bonds, while the amino and carboxyl groups of polyglutamate also exist, so polyglutamate may also cross-link with keratin, which further improves the mechanical properties of ENSs.

### 3.3. Resistance to Hydrolysis

The prepared γ-PGA/keratin blend nanofibers were soluble in water due to their excellent water solubility, which greatly limits their application in biomedicine. In order to solve this problem, it is of great importance to conduct cross-linking treatments on scaffolds to provide the fibers with good water stability. Therefore, the γ-PGA/keratin fiber membranes were cross-linked by using an EDC/NHS/cystamine methanol solution in order to provide the cross-linked γ-PGA/keratin blend fiber membranes with water resistance.

**Figure 3:**
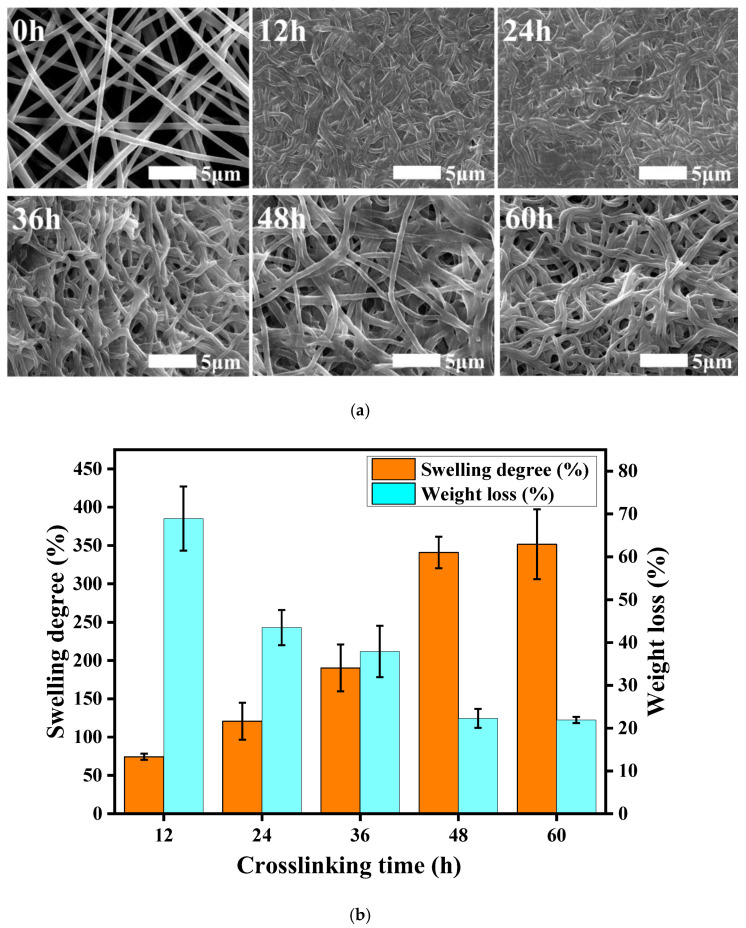
(**a**) SEM images of γ-PGA/keratin fiber membranes at different treatment times; (**b**) swelling degree and weight loss of ENSs under different cross-linking times.

Figure 3a shows SEM images of the fiber membranes after 48 h of immersion in PBS under different cross-linking treatment durations. When the cross-linking time was short (12 h, 24 h), the cross-linking was incomplete, and the fibers partially swelled and stuck together, thereby increasing the fiber diameter and the density with almost no gap. However, after 48 and 60 h of cross-linking, the fibers almost maintained their original morphology and the fiber diameter did not change much. This shows that a cross-linking reaction time of more than 48 h was able to make the γ-PGA/keratin blend nanofibers hydrolysis-resistant, and the fiber morphology did not change significantly.

The cross-linked fiber membranes have a certain anti-hydrolysis property, which can be seen in Figure 3b. With the extension of the cross-linking time, the cross-linking reaction was gradually completed, and the dissolution rate of the membranes showed a trend of gradually decreasing from the original 68.9% to about 22%. When the cross-linking time reached 48 h, the dissolution rate of the fiber membranes was stable at about 22%, which meant that the cross-linking reaction had been completed after 48 h of treatment, and the fiber membrane had good hydrolysis resistance. As the cross-linking time increased, the swelling degree of the membranes also increased gradually, from 74.3% after 12 h of treatment to about 320%. This is because the swelling degree of the fiber membranes was affected by the porosity. When the processing time was shorter, the cross-linking reaction did not finish. The fiber membranes had some swelling synechia during the process of being soaked in water, which resulted in a decrease in the void fraction of the membranes and the space for retaining moisture; thus, the swelling degree was low. In addition, partial dissolution of the fiber membranes can also lead to a lower degree of swelling. As the cross-linking time was extended, the fiber adhesion phenomenon gradually reduced, and the water stability of the membranes also increased, which caused the swelling degree to increase gradually. The swelling performance of the membranes represents the water absorption and water retention capacity of the membranes. The better the swelling performance of the membrane, the more effective it will be when transplanted into the body to retain nutrients and provide favorable conditions for the growth of cells.

**Figure 4:**
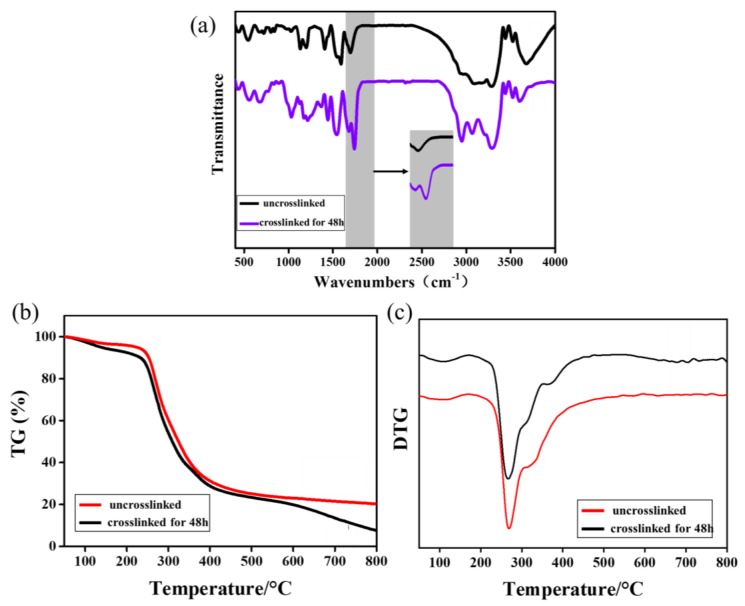
(**a**) FTIR spectra of γ-PGA/keratin before and after cross-linking; (**b**) TG of ENSs before and after cross-linking; (**c**) DTG of ENSs before and after cross-linking.

FTIR spectra of γ-PGA and keratin blend membranes before and after cross-linking are displayed in Figure 4a. The absorption peak near 3290 cm^−1^ was attributed to the overlapping of N-H and O-H stretching vibrations. The absorption peaks at 1676 cm^−1^ and 1540 cm^−1^ in the spectral picture are, respectively, the vibration absorption peaks of amide I and amide II bands [42]. It can be observed from Figure 4a that a new characteristic absorption peak of the cross-linked fiber membrane appears at 1740 cm^−1^, which is the characteristic absorption peak of the amide I band [43]. The reason for this is that the γ-PGA and amino groups in the keratin react with the carboxyl groups under the action of EDS/NHS and cystamine. The changes in the FTIR spectra clearly indicate that EDC/NHS/cystamine was successfully cross-linked with γ-PGA and keratin. 

As can be seen from Figure 4b, the weight loss rate of the ENS is about 5% in the range of 50 °C to 100 °C, which was caused by the evaporation of water in the fiber membrane due to the increase in the temperature. The weight loss of the keratin and γ-PGA was mainly concentrated in the range of 250–500 °C, and the weight loss rate was higher (about 70%). The maximum decomposition rate of the fiber membrane after cross-linking was increased from 260 °C to 270 °C compared with the untreated membrane (Figure 4c). The reason for this is that the cross-linking treatment caused new covalent bonds to be formed between the molecules so that the thermal stability of the fiber membranes was improved. The thermogravimetric test results further confirm the occurrence of the cross-linking reaction.

### 3.4. Tensile Properties

**Figure 5:**
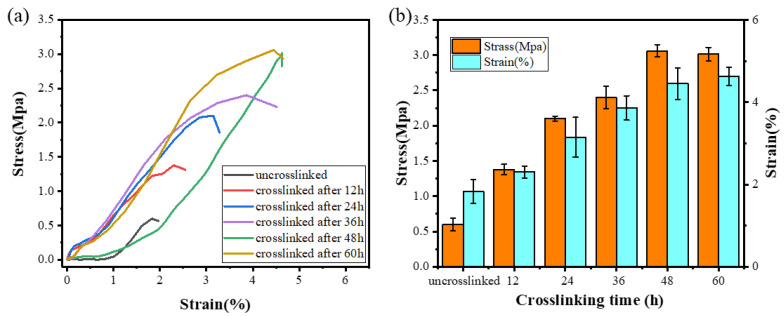
(**a**) Stress–strain curves of the γ-PGA/keratin ENS under different cross-linking times. (**b**) Maximum stress and breaking elongation of the γ-PGA/keratin ENS under different cross-linking times.

In order to further verify the completion time of the cross-linking reaction and evaluate whether its mechanical properties can satisfy the requirements of practical applications, the mechanical properties of ENSs with different cross-linking treatment times were tested. The stress–strain curve of the ENS is shown in Figure 5a. As the cross-linking time increased, the tensile strength of the ENS gradually increased from 0.5 Mpa to 3 Mpa. This is because new covalent bonds were generated during the cross-linking process, indicating that the mechanical properties of the membrane can be effectively improved by the cross-linking method. There was no significant change in the tensile strength and stress of the ENS at 48 h and 60 h of cross-linking (Figure 5b), which further confirms that the ENS cross-linking reaction was basically complete at 48 h.

### 3.5. In Vitro Degradation

**Figure 6:**
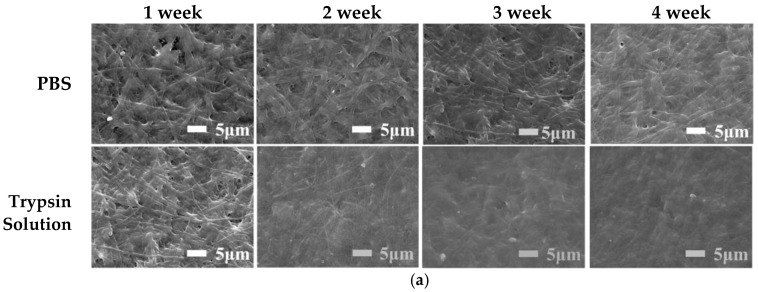

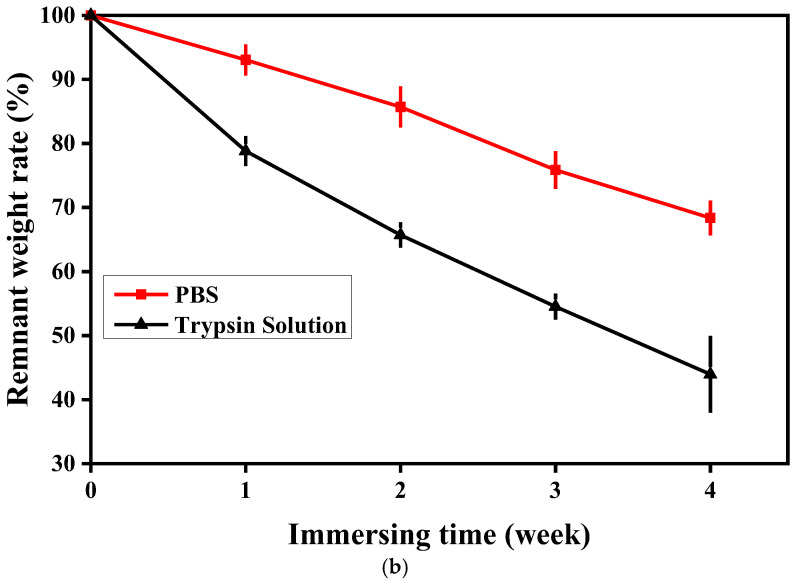
SEM images and weight loss rates of the γ-PGA/keratin ENS immersed in PBS solution and trypsin solution for different times: (**a**) SEM images; (**b**) weight loss rates.

Figure 6 shows the weight of and morphological changes in the ENS during degradation in PBS solution and trypsin solution. As the degradation time increased, the membrane degraded gradually. The original appearance of the ENS disappeared and the fibres adhered to each other after the ENS was degraded in PBS solution and trypsin solution for one week. This phenomenon became more obvious as the degradation time increased (Figure 6a). The degradation rate of the ENS in trypsin solution was higher than that in PBS solution (Figure 6b). During the fourth week of degradation, the residual weight of the ENS in PBS solution was 68.37%, and that in trypsin solution was 43.96%. The reason for this might be that the ENS is composed of γ-PGA and keratin, and trypsin can catalyze the degradation of keratin. After cross-linking, new covalent bonds were formed between the molecules of the membrane, so the degradation rate was low in PBS. It can be seen from Figure 6b that the degradation rate of the ENS is extremely stable in both the PBS solution and the trypsin solution.

### 3.6. Biocompatibility

**Figure 7:**
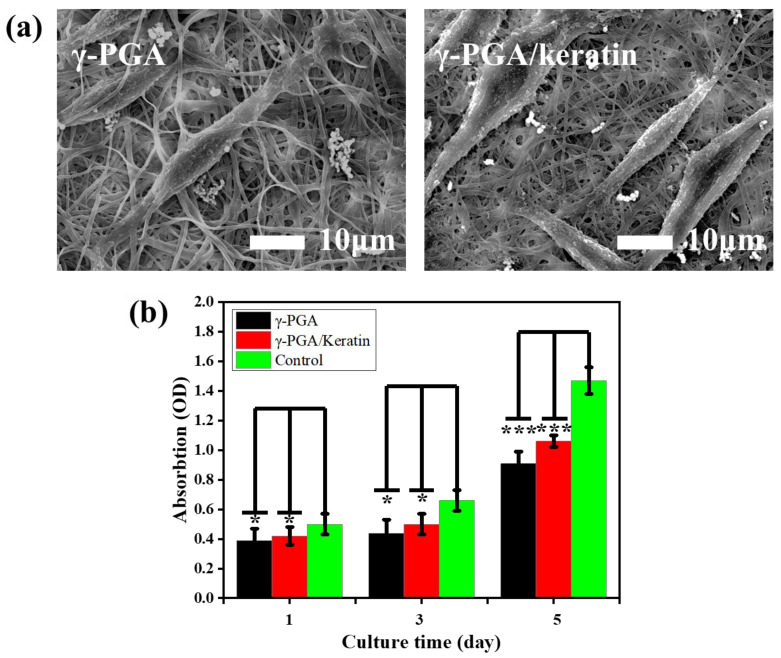
(**a**) SEM images of cells on γ-PGA and γ-PGA/keratin membranes after 3 days of culture. (**b**) Cell viability results of L929 fibroblasts on γ-PGA and γ-PGA/keratin membranes (mean ± SDs; significant difference: * *p* < 0.05, *** *p* < 0.01).

**Figure 8:**
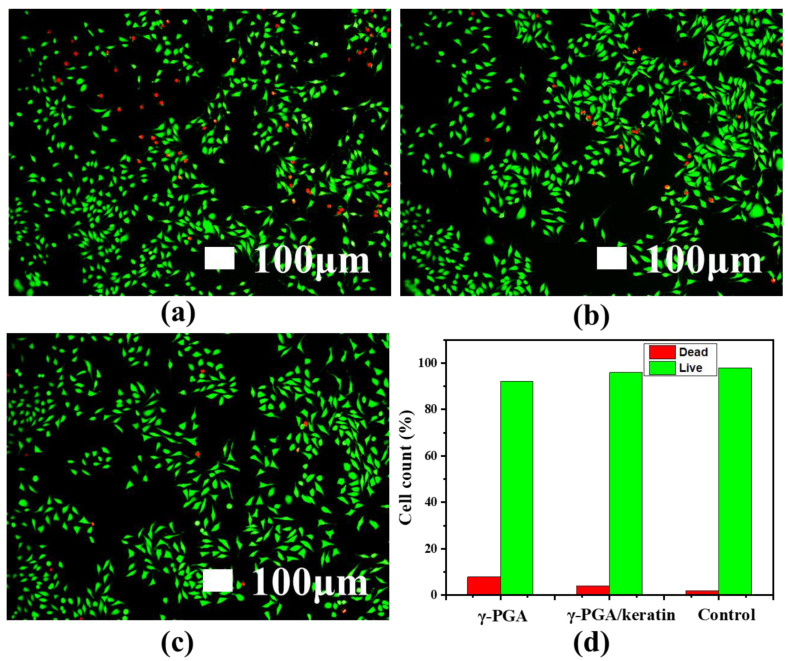
Fluorescence microscopy images of L929 mouse fibroblasts cultured for 1 d: (**a**) γ-PGA; (**b**) γ-PGA/keratin; (**c**) control; (**d**) the corresponding percentage of live/dead cells.

The adhesion of cells on γ-PGA and the γ-PGA/keratin ENS, respectively, after 3 days of culture is shown in Figure 7a. It was observed that the cells formed pseudopodia and attached to the ENS, indicating that the cells grew well on the ENS. This phenomenon suggests that cells can stick to the membranes and the membranes have good cell compatibility. Figure 7b shows the cell proliferation of mouse fibroblasts cultured on γ-PGA and the γ-PGA/keratin ENS, respectively, for 1, 3, and 5 days. The cells were able to grow and proliferate on the γ-PGA/keratin ENS. The γ-PGA/keratin membrane was more suitable for the proliferation of cells compared with the pure γ-PGA membrane. Over time, the relative survival rate of mouse cells on the γ-PGA/keratin ENS and the γ-PGA ENS increased. The survival rates of the γ-PGA/keratin ENS and the γ-PGA ENS are compared in Figure 8. The microscopic images show dead cells both in the fibrous membrane and in the control group. A counting method was used to calculate the percentage of living and dead cells. When the cells were cultured for 1 day, the percentage of dead cells on the γ-PGA membrane was 7.86%, that on the γ-PGA/keratin membrane was 3.95%, and that on the control membrane was 1.95% (Figure 8d). It can be seen that the survival rate of cells on the γ-PGA/keratin ENS was higher, indicating that the biocompatibility of the γ-PGA/keratin ENS was better than that of the γ-PGA ENS. It can be inferred that the addition of keratin can increase the biocompatibility of the membranes.

## 4. Conclusions

In this work, the relationship between electrospinning parameters and the morphology of γ-PGA/keratin nanofibers was systematically studied, and γ-PGA/keratin ENSs were successfully prepared. Under optimized spinning parameters, smooth and uniform γ-PGA/keratin nanofibers were successfully prepared for the first time with an average diameter of 554 ± 73 nm. In addition, the principle of the cross-linking reaction was studied. The cross-linked γ-PGA/keratin ENS was provided with excellent water stability by using the EDC/NHS/cystamine methanol solution. The cross-linked γ-PGA/keratin ENS was stably degraded in both PBS solution and trypsin solution. Moreover, in vitro cell experiments showed that the γ-PGA/keratin ENS can promote cell proliferation and adhesion. The addition of keratin can improve the biocompatibility of the membrane. The γ-PGA/keratin ENS has great application prospects in the field of tissue engineering.

## Data Availability

Data sharing is not applicable to this article.
